# The complete chloroplast genome sequence of *Nepeta cataria L*. (Labiatae)

**DOI:** 10.1080/23802359.2020.1813058

**Published:** 2020-09-01

**Authors:** Hui Zhou, Linbo Du, Zhihong Fang

**Affiliations:** Department of Pediatrics, Suizhou Hospital, Hubei Medical College, Suizhou, China

**Keywords:** *Nepeta cataria*, Labiatae, complete chloroplast genome

## Abstract

*Nepeta cataria L.* is a common herbal medicine and it has been applied to the treatment of various diseases in China, especially common cold in children. In this study, we reported and characterized the complete chloroplast genome sequence of *N. cataria L.* The chloroplast genome was determined to be 152,339 bp in length. It contained large single-copy (LSC) and small single-copy (SSC) regions of 95,821 bp and 23,460 bp, respectively, which were separated by a pair of 16,529 bp inverted repeat (IR) regions. The genome is predicted to contain 132 genes, including 87 protein-coding genes, 37 tRNA genes, and 8 rRNA genes. The overall GC content of the genome is 37.85%. The complete plastome sequence of *N. cataria L.* will provide a useful resource for the conservation genetics of this species as well as for the phylogenetic studies for *Schizonepeta tenuifolia Briq.* A phylogenetic tree reconstructed by 19 chloroplast genomes reveals that *N. cataria L.* is mostly related to *Callicarpa nudiflora*, but forms an independent evolutionary branch.

*Nepeta cataria L.* is the dry overground part of *Schizonepeta tenuifolia Briq.* and it is one of the eight Chinese Qi medicines in China. Its original name is Stachyopsis, or known as fragrant *N. cataria L.*, *Cardamine* and *Artemisia quadrangularis*. Currently, *N. cataria L.* is produced in Xinjiang, Gansu, Shaanxi, Henan, Shanxi, Shandong, Hubei, Guizhou, Sichuan and Yunnan. Internationally, *N. cataria L.* is distributed from central and south Europe to Japan through Afghanistan and there are wild resources in America and south of Africa (Herron 2003). However, there is no complete chloroplast genome characteristics of *N. cataria L.* available yet. Therefore, sequencing of *N. cataria L.* based on two-end sequencing of Illumina was performed and the first complete chloroplast genome sequence was gained and identified.

Whole-genome of specimens were collected from the Hubei University of Medicine Affiliated Suizhou Hospital (N39°91′09.25ʺ, E116°41′33.84ʺ). The plant DNA Mini Kit (geneponeer, Nanjing) was used to extract total genome DNA from fresh leaves of *N. cataria L.* and samples were stored in Hubei University of Medicine Affiliated Suizhou Hospital (Number of Certificate: N2967). Whole-genome sequencing was performed by Nanjing Genepioneer Biotech Company (Nanjing, China) on the IlluminaHiseq platform. The filtered sequences were assembled by the Spades programming 3.10.0 (Bankevich et al. 2012). CpGAVAS was used for annotation (Liu et al. 2012) and BLAST searching was implemented.

Results demonstrated that the chloroplast genome of *N. cataria L*. is composed of 152,339bp double-stranded DNAs and it contains two 16,529 bp inverted repeat (IR) regions. These two IR regions were separated by 95,821 bp large single-copy (LSC) region and 23,460 bp small single-copy (SSC) region. Chloroplast genome is expected to contain 132 genes, including 87 protein-coding genes, 7 tRNA genes and 4 rRNA genes. The 21 genes contain 1 intron and 2 genes (clpP and ycf3) contain 2 introns. Total GC content of chloroplast genome of *N. cataria L*. is 37.85%, and values of LSC, SSC, and IR regions are 35.90%, 31.65%, and 43.15%, respectively.

To study the systematic evolution of *N. cataria L*., the chloroplast genome sequences of 18 plants and chloroplast genome sequences of *N. cataria L*. were chosen for multiple sequence alignment by using MAFFT v7.307 software (Katoh and Standley 2013). The comparison data were trimmed by trimAl (v1.4. rev 15) (Fedosova and Kovalenko 2015). Next, a rapid bootstrap analysis was conducted by using RAxML v8.2.10 and the GTRGAMMA model, with bootstrap = 1000. The maximum likelihood evolutionary tree was constructed (*Dracocephalum palmatum* and *Glechoma longituba* were used as the outgroup) (Alexandros et al. 2008). Analysis results are shown in Figure 1. According to the phylogenetic analysis, *N. cataria L*. forms an independent branch compared to other plants, which is related to the genetic relation of *N. cataria L*. A complete chloroplast genome sequence of *N. cataria L*. provides studies for genetic resources to protect *N. cataria L*.

**Figure 1.**
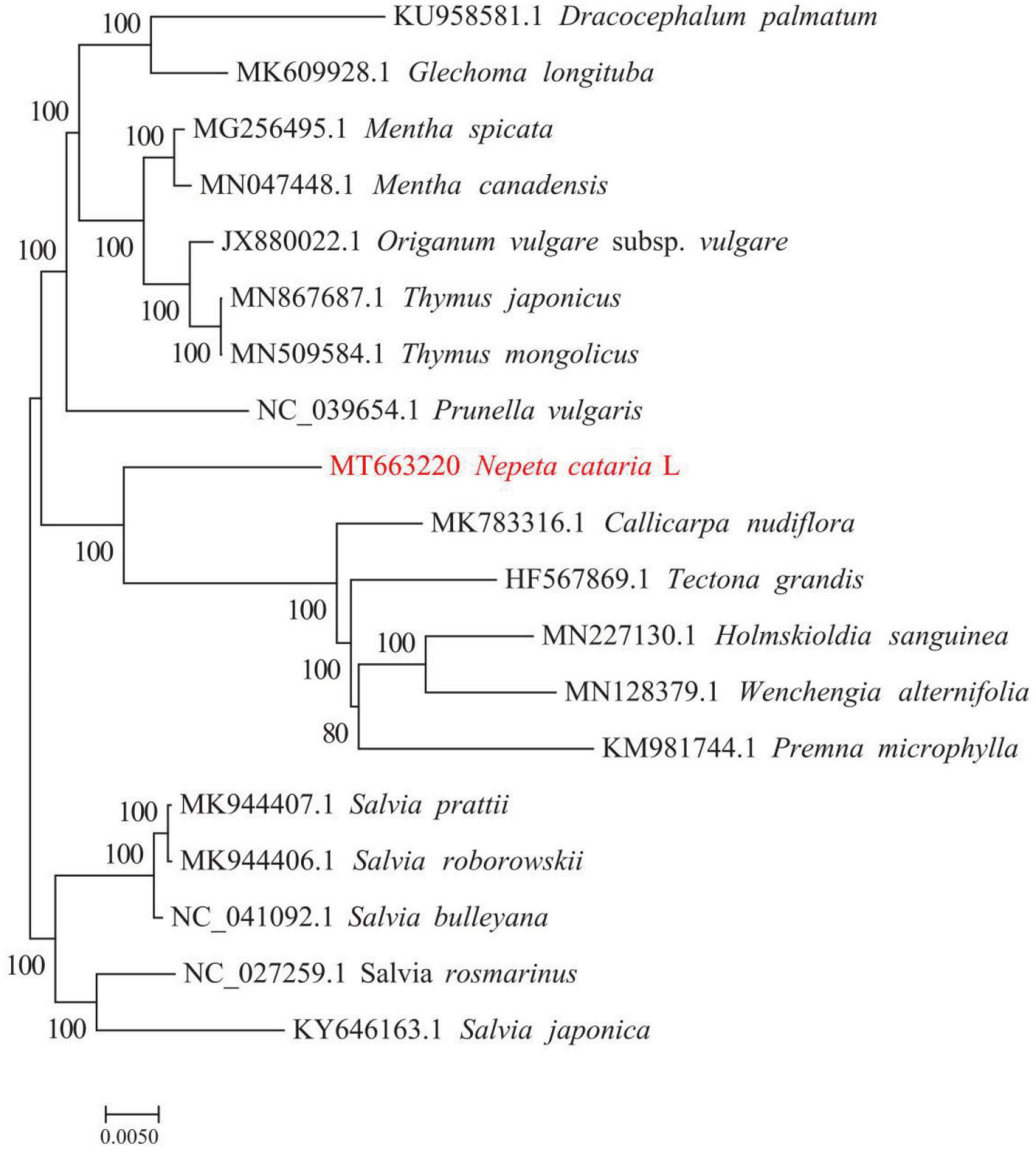
Phylogenetic tree plotting using maximum likelihood (ML) method based on the complete chloroplast genome of *Nepeta cataria L*. and 18 representative species. The bootstrap value is displayed on branches.

## Data Availability

The data that support the findings of this study are openly available in GenBank of NCBI at https://www.ncbi.nlm.nih.gov, reference number MT663220.
